# Skeletal Muscle Fiber Type in Hypoxia: Adaptation to High-Altitude Exposure and Under Conditions of Pathological Hypoxia

**DOI:** 10.3389/fphys.2018.01450

**Published:** 2018-10-12

**Authors:** Thomas Chaillou

**Affiliations:** School of Health Sciences, Örebro University, Örebro, Sweden

**Keywords:** myosin heavy chain, oxygen, hypoxia-inducible factor-1 alpha, chronic obstructive pulmonary disease, chronic heart failure, obstructive sleep apnea syndrome, muscle plasticity

## Abstract

Skeletal muscle is able to modify its size, and its metabolic/contractile properties in response to a variety of stimuli, such as mechanical stress, neuronal activity, metabolic and hormonal influences, and environmental factors. A reduced oxygen availability, called hypoxia, has been proposed to induce metabolic adaptations and loss of mass in skeletal muscle. In addition, several evidences indicate that muscle fiber-type composition could be affected by hypoxia. The main purpose of this review is to explore the adaptation of skeletal muscle fiber-type composition to exposure to high altitude (ambient hypoxia) and under conditions of pathological hypoxia, including chronic obstructive pulmonary disease (COPD), chronic heart failure (CHF) and obstructive sleep apnea syndrome (OSAS). The muscle fiber-type composition of both adult animals and humans is not markedly altered during chronic exposure to high altitude. However, the fast-to-slow fiber-type transition observed in hind limb muscles during post-natal development is impaired in growing rats exposed to severe altitude. A slow-to-fast transition in fiber type is commonly found in lower limb muscles from patients with COPD and CHF, whereas a transition toward a slower fiber-type profile is often found in the diaphragm muscle in these two pathologies. A slow-to-fast transformation in fiber type is generally observed in the upper airway muscles in rodent models of OSAS. The factors potentially responsible for the adaptation of fiber type under these hypoxic conditions are also discussed in this review. The impaired locomotor activity most likely explains the changes in fiber type composition in growing rats exposed to severe altitude. Furthermore, chronic inactivity and muscle deconditioning could result in the slow-to-fast fiber-type conversion in lower limb muscles during COPD and CHF, while the factors responsible for the adaptation of muscle fiber type during OSAS remain hypothetical. Finally, the role played by cellular hypoxia, hypoxia-inducible factor-1 alpha (HIF-1α), and other molecular regulators in the adaptation of muscle fiber-type composition is described in response to high altitude exposure and conditions of pathological hypoxia.

## Introduction

Skeletal muscles are composed of heterogeneous fiber types with distinct metabolic and contractile properties. Slow-twitch oxidative (SO) fibers are characterized by high content of mitochondria, myoglobin, and high capillary density. Fast-twitch glycolytic (FG) fibers have lower oxidative capacities, reduced capillary density and usually have bigger cross-section areas, while fast-twitch oxidative glycolytic (FOG) fibers exhibit intermediate properties. More recent research usually classifies skeletal muscle fiber types according to their isoforms of the contractile protein myosin heavy chain (MHC): type-I fibers, type-IIA fibers, and type-IIX/IIB fibers; these types of fibers have similar characteristics as SO, FOG, and FG fibers, respectively. It is noteworthy that type-IIB fibers are absent in human skeletal muscles (Schiaffino, 2010). In addition, two developmental MHC isoforms, embryonic MHC and neonatal MHC, are expressed in skeletal muscle fibers during embryogenesis and neonatal development, as well as during muscle regeneration (Chargé and Rudnicki, 2004). Skeletal muscle has a remarkable ability to modify its size and adjust its metabolic and contractile properties to a variety of stimuli. Skeletal muscle phenotype, which is mainly determined by genetic factors, can be modulated by endogenous and exogenous stimuli, including mechanical strains and neuronal activity (i.e., contractile activity), metabolic and hormonal influences, as well as environmental factors (Flück, 2006).

A reduced level of oxygen (O_2_), called hypoxia, appears in many situations, including exposure to high altitude (ambient hypoxia) (West, 1987), physical exercise (physiological and transient hypoxia) (Richardson et al., 1995) and diseases (pathological hypoxia) (Semenza, 2014). Metabolic adaptations and loss of mass in skeletal muscle have been extensively studied in response to chronic exposure to high altitude and is not the focus of this review (for recent reviews, see Favier et al., 2015; Murray and Horscroft, 2016). Over the past 40 years, numerous studies have evaluated changes in skeletal muscle fiber type in response to severe ambient hypoxia. Some findings have been reported in a review almost 20 years ago by a research group (Ishihara et al., 2000), but this work was restricted to animal studies. In addition, changes in the composition of skeletal muscle fiber type have been observed over the last 25 years in conditions of pathological hypoxia, including diseases related to the cardio-respiratory system such as chronic obstructive pulmonary disease (COPD), chronic heart failure (CHF), and obstructive sleep apnea syndrome (OSAS).

The purpose of this review is to present an overview of the adaptation of skeletal muscle fiber-type composition in response to high altitude exposure and conditions of pathological hypoxia, including diseases of the cardio-respiratory system associated with reduced arterial oxygen saturation (COPD, CHF, and OSAS). Other diseases such as cancer cachexia, which may result in local muscle hypoxia (Devine et al., 2017), will not be included in this review because the exact origin of muscle hypoxia is extremely complex and most likely multifactorial. Then, the factors potentially responsible for the adaptation of skeletal muscle fiber-type composition in hypoxic conditions will be discussed. In addition, the role played by the hypoxia-inducible factor-1 signaling pathway and other molecular regulators in the modulation of skeletal muscle fiber type will be examined. Furthermore, the implication of cellular hypoxic stress in the adaptation of skeletal muscle fiber type to high-altitude exposure and under conditions of pathological hypoxia will be analyzed.

## Adaptation of skeletal muscle fiber type during exposure to high altitude

Exposure to high-altitude environment leads to a reduced O_2_ availability due to a low barometric pressure. The O_2_ pressure (PO_2_) markedly decreases through the transport of O_2_ from the ambient air to the tissues such as skeletal muscles. Due to the low ambient PO_2_ present in high altitude, PO_2_ is consequently reduced in skeletal muscle (Richardson et al., 2006). Ambient hypoxia can be reproduced experimentally by either reducing the barometric pressure without affecting the inspired O_2_ fraction (i.e., hypobaric hypoxia), or by reducing the inspired O_2_ fraction without changing the barometric pressure (i.e., normobaric hypoxia) (Chaillou and Lanner, 2016). Chronic exposure to high altitude has been shown to affect the structural, functional and metabolic profiles of skeletal muscle, including changes in the capillary network, oxidative capacity, and myofiber size (Breen et al., 2008; Favier et al., 2015; Murray and Horscroft, 2016). The impact of chronic exposure to ambient hypoxia on the composition of muscle fiber type has been extensively investigated in rodents while the scientific knowledge remains limited in humans. A summary of the main findings is presented in Table 1.

**Table 1:** Adaptation of skeletal muscle fiber type in response to ambient and simulated hypoxia.

**Species**	**Gender**	**Age**	**Hypoxia expsoure**	**Muscles**	**Main effect**	**References**
Human	M	21–31 y	40 d, 0 → 8,848 m (decompression chamber)	VL	X vs. Before	Green et al., 1989
	M	23 y	3 w at 4,300 m (ambient hypoxia)	VL	X vs. before	Green et al., 1992
		18 y	High-altitude native Tibetans	VL	X vs. Nepalese lowlanders	Kayser et al., 1996
	M	24.2 y	High-altitude natives of La Paz (Bolivia)	VL	No comparison with lowlanders	Desplanches et al., 1996
	M, F	22–31 y	8 w at 4,100 m (El Alto, Bolivia)	VL	X vs. Before	Juel et al., 2003
	M	26–37 y	High-altitude natives of la Paz/El Alto (Bolivia)	VL	X vs. Caucasian lowlanders	
	M	39 y	23 d at > 5,000 m (expedition in the Himalayas)	VL	↑ % FI and ↓ % FIIA, ↑ % MHC1 and ↓ % MHC2A vs. Before	Doria et al., 2011
Rat	F	Developing	6 w at 5,100 m (normobaric hypoxia)	SOL	X vs. CT	Sillau and Banchero, 1977
				GAS, TA	↑ % FII and ↓ % FI vs. CT	
			7 w at 4,000 m (hypobaric hypoxia)	SOL	↑ % FOG and ↓ % SO vs. CT	Itoh et al., 1988
	M	4 w	10 w at 4,000 m (hypobaric hypoxia)	SOL	↑ % FOG and ↓ % SO vs. CT	Itoh et al., 1990
				EDL	↑ % FOG and ↓ % FG vs. CT	
	M	5–6 w	14 w at 4,000 m (hypobaric hypoxia)	SOL	X vs. CT	Bigard et al., 1991
				EDL	↑ % FIIA/IIB vs. CT	
				PLA (deep)	↑ % FIIA/IIB and ↓ % FIIA vs. CT	
	M, F	Born in hypoxia	30 w at 4,000 m (hypobaric hypoxia)	SOL	↑ % FOG and ↓ % SO vs. CT	Hirofuji et al., 1992
	M	5 w	7 w at 4,000 m (hypobaric hypoxia)	SOL	↑ % FOG vs. CT	Itoh et al., 1992
	M	3 w	12 w at 4,000 m (hypobaric hypoxia)	SOL	↑ % FOG and ↓ % SO vs. CT	Itoh et al., 1995
	M	5, 10, and 15 w	5 w at 4,000 m (hypobaric hypoxia)	SOL	↓ % SO and ↓ % MHC1 in younger rats (5 and 10 w) vs. CT	Ishihara et al., 1995
	M	8 w	8 w at 2,250–2,550 m (hypobaric hypoxia)	EDL	X vs. CT	Perhonen et al., 1996
	M	5 and 10 w	4,000 m (hypobaric hypoxia): 5 w for younger rats, 10 w for older rats	EDL	↑ % FOG and ↓ % FG in younger rats vs. CT, X in older rats vs. CT	Ishihara et al., 2000
	M	3–4 w	4 w at 5,500 m (hypobaric hypoxia)	SOL	↑ % FIIA and ↓ % FI, ↑ % MHC2A and ↓ % MHC1 vs. CT	Bigard et al., 2000
				PLA	↓ % FI and FIIA, ↑ % FIIB vs. CT	
	M	5–6 w	3 w at 4,000 m (normobaric hypoxia)	SOL, EDL, TA	X vs. CT	Deveci et al., 2001
	M	11 w	4 w at 5,500 m (normobaric hypoxia)	SOL	↑ % FIIA and ↓ % FI vs. CT	Faucher et al., 2005
				EDL	↓ % FIIX vs. CT	
	F	8–9 w	53 d at 5,500 m (hypobaric hypoxia)	PLA	X vs. CT	Chaillou et al., 2013
	F	8–9 w	35 d at 5,500 m (hypobaric hypoxia)	SOL	X vs. CT	Chaillou et al., 2014
	M	7 w	7.5 h/d (11.5% O_2_) for 2 d or 4 d (normobaric hypoxia)	SOL	X vs. CT	Nguyen et al., 2016
				TA	X vs. CT	
				GH	In single fibers: ↓% MHC2A, ↑% MHC2A/2B, and ↑% MHC2B vs. CT	
Mouse	M	4, 12, and 52 w	3 w at 8% O_2_ (normobaric hypoxia)	SOL	↑ % FI/IIA and ↓ % FIIA in young mice vs. CT	Slot et al., 2016
				EDL	X vs. CT	
	M	8–10 w	4 w at 10 % O_2_ (normobaric hypoxia)	SOL	↓ % FIIX and FIIB vs. CT	Shin et al., 2016
				GAS	↑ % FIIA, ↓ % FIIX and FIIB vs. CT	
			High-altitude native mice	GAS	↑ % FI vs. low-altitude native mice	Scott et al., 2015
	M, F	Adult	High-altitude native mice bred and raised in N	GAS	↑ % FI vs. low-altitude native mice	Lui et al., 2015
			6–8 w at 4,300 m (hypobaric hypoxia)	GAS	X vs. CT	
	M, F	Adult	High-altitude native mice bred and raised in N	Core GAS	X vs. low-altitude native mice	Mahalingam et al., 2017
			6–10 w at 4,300 m (hypobaric hypoxia)	Core GAS	↓ % FI vs. CT	
	M, F	6–12 mo	High-altitude native mice bred and raised in N	DIA	X vs. low-altitude native mice	Dawson et al., 2018
			6–8 w at 4,300 m (hypobaric hypoxia)	DIA	↓ % FIIA vs. CT	
	M	7 w	4 w at 10 % O_2_ (normobaric hypoxia)	SOL	↑ % FII and ↓ % FI vs. CT	O'Brien et al., 2018

*M, male; F, female; y, year; mo, month, w, week; d, day; m, meter; N, normoxia; VL, vastus lateralis muscle; SOL, soleus muscle; GAS, gastrocnemius muscle; TA, tibialis anterior muscle; EDL, extensor digitorum longus muscle; PLA, plantaris muscle; GH, geniohyoid muscle; DIA, diaphragm muscle; X, not significantly different; ↑, increase; ↓, decrease; CT, control group; FI, type-I fiber; FIIA, type-IIA fiber; FIIA/IIB, type-IIA/IIB fiber; FIIX, type-IIX fiber; FIIB, type-IIB fiber; SO, Slow-twitch oxidative fiber; FOG, fast-twitch oxidative glycolytic fiber; FG, Fast-twitch glycolytic fiber; MHC1, myosin heavy chain 1; MHC2A, myosin heavy chain 2A; MHC2A/2B, myosin heavy chain 2A/2B; MHC2B, myosin heavy chain 2B. The age of the animals presented in the table corresponds to the age at the beginning of the experiments*.

### Hypoxia-induced muscle fiber-type adaptation in animals: effect of muscle characteristics, age and gender

Several early studies performed on rats observed a faster fiber-type profile in response to severe ambient hypoxia (simulated altitude > 4,000 m) compared with normoxia (Itoh et al., 1988, 1990, 1995; Bigard et al., 1991, 2000; Hirofuji et al., 1992; Ishihara et al., 1995; Faucher et al., 2005). In contrast, no changes in muscle fiber-type composition seem to occur in moderate hypoxia (<3,000 m) (Perhonen et al., 1996). For instance, the proportion of FOG fibers was significantly higher in slow-oxidative soleus muscles from hypoxic rats exposed to a simulated altitude of 4,000 m for 7 weeks compared with normoxic rats (28.8 and 19.2%, respectively), while the opposite result was observed for the proportion of SO fibers (Itoh et al., 1988). A lower percentage of type-IIA fibers and a higher percentage of hybrid type-IIAB fibers have been reported in the deep portion of the fast glycolytic plantaris muscles from hypoxic rats after 14 weeks at 4,000 m compared with normoxic animals (Bigard et al., 1991). A faster fiber-type profile in the plantaris muscles of hypoxic rats compared with control rats has been confirmed a few years later by the same group (Bigard et al., 2000). However, the hypoxia-induced changes in muscle fiber-type composition in the soleus muscle (Sillau and Banchero, 1977; Bigard et al., 1991; Deveci et al., 2001; Chaillou et al., 2014; Slot et al., 2016) and in fast glycolytic muscles (Itoh et al., 1990; Ishihara et al., 2000; Deveci et al., 2001; Chaillou et al., 2013; Slot et al., 2016) remain questioned. Several factors can be proposed to explain these discrepancies. One explanation may be the methods performed to determine the fiber-type composition (enzyme histochemical analysis, immunohistochemical analysis using antibodies against MHC, or electrophoresis of MHC isoforms). Another reason that could potentially lead to divergences in studies focusing on fast glycolytic muscles is the distinct functions between the plantaris muscle (i.e., ankle extensor) and extensor digitorum longus (EDL) muscle (i.e., ankle flexor). Finally, we believe that in addition to the animal species (e.g., rat or mouse), the age of the animals highly contributes to explain these controversial results.

In a recent study, we did not observed any changes in the proportion of MHC isoforms in soleus muscles from growing rats (8–9 weeks old at the start of the experiments) exposed 35 days at a simulated altitude of 5,500 m (Chaillou et al., 2014). However, a marked adaptation of MHC isoform composition in response to ambient hypoxia was observed in younger rats (3–4 weeks old before hypoxia exposure) in a previous study from our group (Bigard et al., 2000). It has been proposed that chronic exposure to altitude impairs the fast-to-slow fiber-type transition which appears in soleus muscles from growing rats during post-natal development (Ishihara et al., 2000). In a study published in 1995, growing male Sprague-Dawley rats of 5, 10, and 15 weeks of age were exposed to hypobaric hypoxia (equivalent to 4,000 m) for 5 weeks, while control animals of identical ages were maintained in normoxia (Ishihara et al., 1995). It was demonstrated that ambient hypoxia inhibits the fast-to-slow shift in fiber-type and MHC isoform composition observed in normoxia in the youngest rats (5 and 10 weeks old before hypoxia exposure). In contrast, hypoxia had no effect on the profile of fiber types and MHC isoforms in 15 week-old rats, most probably because the mature profile was already established at this age. In our study, hypoxia did not affect the MHC isoform composition of the soleus muscle in female Wistar Han rats that were only 8–9 weeks old before hypoxia exposure (Chaillou et al., 2014). This discrepancy between the latter study and the study from the Japanese group (Ishihara et al., 1995) may result from the strain and the gender of the rats.

The effect of gender has been investigated in second-generation hypoxia-acclimated rats bred in hypobaric hypoxia (4,000 m) (Hirofuji et al., 1992). Soleus muscles were collected at 3, 5, 8, 10, and 30 weeks of age and the fiber-type composition was compared to age-matched normoxic rats. The fiber-type shift that occurs in soleus muscles during postnatal development in normoxia was blunted in male hypoxic rats. In female hypoxic rats, the proportion of FOG fibers decreased during development, but remained higher than that observed in age-matched normoxic animals. To date, the gender-related influence on the adaptation of fiber type in growing rats exposed to severe hypoxia remains to be elucidated. Several factors could be hypothesized, such as a gender-specific adaptation of voluntary locomotor activity and food intake during ambient hypoxia, or a gender-specific sensitivity to hypoxia exposure. In addition, the endocrine secretion of hormones such as thyroid hormones, which appears to be affected in high altitude (Connors and Martin, 1982), might be another potential factor.

### Hypoxia-induced muscle fiber-type adaptation in animals: effect of food intake and voluntary physical activity

As presented in the part above, cumulative evidences indicate that prolonged exposure to severe hypoxia impairs the fast-to-slow fiber-type shift in soleus muscles during rat postnatal development. However, it has been questioned whether these fiber-type changes were the result of either hypoxia *per se* (i.e., reduced O_2_ availability), reduced food intake or lowered voluntary locomotor activity (i.e., contractile activity) consecutive to hypoxia exposure (Bigard et al., 2000; Chaillou et al., 2013).

A reduction of daily food intake is usually observed in growing rats exposed to severe hypoxia (~30% during the first 4 weeks at a simulated altitude of 5,500 m) compared with normoxic rats (Bigard et al., 2000; Chaillou et al., 2012). In order to control the potential impact of hypoxia-induced hypophagia, a group of normoxic animals paired-fed with an equivalent quantity of food than that consumed by hypoxic animals was included in the study (Bigard et al., 2000). No changes in fiber-type composition were observed in paired-fed animals in comparison with normoxic animals fed *ad libitum*, demonstrating that fiber-type changes in hypoxia cannot be attributed to the decreased energy intake.

A low level of voluntary locomotor activity (assessed from the wheel running distance) has been shown in growing rats exposed to ambient hypoxia (Bigard et al., 2000), suggesting that the lower muscle contractile activity could contribute to explain the fiber-type changes in hypoxia. In a recent study, we used the model of functional overload of the plantaris muscle induced by the synergist ablation of the soleus and gastrocnemius muscles (Chaillou et al., 2013). This rodent model is relevant in this context because (1) it induces a prolonged activation and recruitment of the plantaris muscle, (2) it can potentially prevent the negative effect of hypoxia on the contractile activity, and (3) it results in a fiber-type shift toward a slower phenotype (Chaillou et al., 2013). Our results indicated that overload-induced fast-to-slow transition in MHC isoforms was only slightly minimized by hypoxia during the early phase of exposure, and the overload-induced changes in MHC isoform composition were identical in normoxic and hypoxic animals after 56 days of mechanical overload.

Recently, we have used another remodeling model to mimic the fast-to-slow fiber-type transition in rat soleus muscle (Chaillou et al., 2014), a muscle suggested to be more sensitive to reduced O_2_ availability (Ishihara et al., 2000). The model of notexin-induced soleus muscle regeneration leads to the re-expression of embryonic and neonatal MHC isoforms, followed by the expression of fast MHC isoforms, and finally by the recovery of the slow MHC profile (Chaillou et al., 2014). Since a full recovery of functional innervation is determinant for the restoration of the slow phenotype of regenerated muscles, we believed that this model was relevant because the neural influences mediated by the regenerative process could potentially exceed the deleterious effect of hypoxia on contractile activity (i.e., neuromuscular activity). Similarly to our previous study (Chaillou et al., 2013), hypoxia did not markedly influence the recovery of the slow MHC profile in regenerated soleus muscles. It only slightly increased the proportion of embryonic-MHC isoform after 7 days of regeneration compared with normoxia, while a moderate reduction in the proportion of neonatal-MHC isoform was found at the same time point. However, the MHC isoform profile was similarly recovered in both normoxia and hypoxia after 28 days of regeneration.

Altogether, these two recent studies reinforce the idea that ambient hypoxia-induced alteration in the fast-to-slow fiber-type shift in soleus muscles during postnatal development is probably not the result of reduced O_2_ availability. It appears more evident that the decreased locomotor activity consecutive to severe hypoxia is the main factor responsible for fiber-type changes in growing animals exposed to a severe hypoxic environment.

### Hypoxia-induced muscle fiber-type adaptation in low-altitude native humans

The adaptation of skeletal muscle fiber-type composition in humans during chronic exposure to severe hypoxia has been investigated in a limited number of studies. In a work published in 1989, called “Operation Everest II,” young males were exposed to progressive hypobaric hypoxia (reaching a simulated altitude of 8,848 m) in a decompression chamber during 40 days (Green et al., 1989). The biopsies from the vastus lateralis (VL) muscle did not reveal any changes in the fiber-type distribution after hypoxia exposure compared with the initial measurements. This result was confirmed by others in environmental hypoxia (3,700–4,100 m above sea level for 8 weeks) (Juel et al., 2003). In a more recent study, seven male climbers participated in a 43-day expedition in the Himalayas, with 23 days spent at high altitude (>5,000 m) (Doria et al., 2011). The percentage of type-I fibers significantly increased after compared with before the expedition (52.2 and 34.7%, respectively), while a reduced proportion of type-IIA fibers was observed after the expedition (before: 31.9%, after: 19.6%). It has been demonstrated that the combination of physical exercise with hypoxia (simulated altitude of 4,000 m) results in a higher increase in the density of muscle subsarcolemmal mitochondria compared with training in normoxia, a result probably explained by a more severe metabolic stress (Schmutz et al., 2010). In the latter study, it is believed that the large volume of physical aerobic exercise is the major factor responsible for the shift in muscle fiber type in the high-altitude trekkers (Doria et al., 2011).

### Muscle fiber-type in high-altitude native animals and humans

Most of our understanding of skeletal muscle adaptation in response to ambient hypoxia derives from studies from animals or humans who are native to lowland environment. Numerous vertebrates, including birds, rodents and humans reside in high-altitude environment. Living in high altitude has led to genetic and physiological modifications in order to adapt to this extreme environment (Storz et al., 2010).

Some animal studies have reported that bar-headed goose, a species which migrates biannually over the Himalayas between southern and central Asia, had a larger proportion of oxidative fibers in the pectoralis major muscle in comparison with that of low-altitude geese (Scott et al., 2009). It is noteworthy that the birds used in this study were bred and raised in captivity at low altitude, and all species had a close phylogenetic relationship. This indicates that the bar-headed geese have evolved and adapted their muscle phenotype to sustain prolonged exercise in extreme hypoxia. Some recent studies have examined the changes in skeletal muscle phenotype that have occurred in high-altitude native deer mice (Lui et al., 2015; Scott et al., 2015; Mahalingam et al., 2017; Dawson et al., 2018). This rodent model is relevant to study this evolutionary adaptation because deer mice live within a range of altitude from below sea level to ~4,300 m above sea level. The fiber-type distribution of the diaphragm and the core gastrocnemius muscles was similar in both high-altitude and low-altitude native deer mice bred and raised in captivity (i.e., in normoxia) (Mahalingam et al., 2017; Dawson et al., 2018). In contrast, two studies have shown that the proportion of type-I fibers in gastrocnemius muscle was larger in high-altitude than low-altitude native deer mice (Lui et al., 2015; Scott et al., 2015). This evolutionary adaptation may be the result of the high metabolic demands required to support locomotion and thermogenesis under conditions of severe hypoxic and cold stress.

Chronic exposure to severe ambient hypoxia does not appear to modify muscle fiber-type composition in adult humans living at low altitude (i.e., lowlanders), at least when strenuous physical training is not coupled with hypoxia exposure. High-altitude natives such as residents from the Himalayas (Tibetans and Nepalese Sherpas), the South American Andes (Quechuas) and the Ethiopian Highlands, have an extraordinary tolerance to hypoxia (for review, see Gilbert-Kawai et al., 2014). Adaptations of muscle ultrastructural properties (fiber size, capillary density, mitochondrial content) and metabolism have been reported between high-altitude natives (Sherpas) and Caucasian lowlanders (Kayser et al., 1991; Horscroft et al., 2017). However, the fiber-type composition is broadly similar between high-altitude natives and lowlanders. The proportion of type-I fibers was similar (~55–60%) in the VL muscle from both Nepalese lowlanders and high-native Tibetans residents (Kayser et al., 1996). A similar percentage was observed by others in type-I fibers from Bolivian high-altitude natives, a result which was not different when compared with Caucasian lowlanders (Juel et al., 2003). Further research with more advanced histochemical analyses and a larger number of subjects is required to clarify whether the fiber-type composition is modified in high-altitude native human populations.

## Adaptation of skeletal muscle fiber type under conditions of pathological hypoxia

### Adaptation of skeletal muscle fiber type in COPD

COPD, a progressive disease mainly caused by smoking, is characterized by a reduced maximal expiratory flow (Faulkner et al., 2006). The air obstruction and difficulties with breathing observed in COPD patients are commonly associated with hypoxemia, which potentially can result in local tissue hypoxia (Wüst and Degens, 2007). Muscle dysfunction, including muscle atrophy, loss of strength and increased fatigue is a common hallmark observed in patients with COPD (for review, see Gea et al., 2013). Changes in muscle fiber-type composition is usually found in these patients compared with control subjects, but the adaptation of fiber type in COPD is different between peripheral and respiratory muscles.

Slow-to-fast fiber type transition is a common feature in lower limb skeletal muscles from COPD patients (Table 2). This change is characterized by a reduced proportion of type-I fibers and an increased proportion of type-II fibers in the VL muscles from patients with severe COPD compared with control subjects (Hildebrand et al., 1991; Whittom et al., 1998; Maltais et al., 1999; Thériault et al., 2014; van de Bool et al., 2016; Ausin et al., 2017). In contrast, this slow-to-fast shift in fiber type is not found in the VL muscle from subjects with mild-to-moderate COPD (Doucet et al., 2004), while this transition does not seem to exist in upper peripheral muscles such as the deltoid muscle (Gea et al., 2001). Several factors have been proposed to explain the skeletal muscle dysfunction in COPD, such as muscle deconditioning and reduced physical activity, neuromuscular degeneration, systemic oxidative stress and inflammation, exacerbated catabolism, hormonal disturbance, smoking, nutritional status, pharmacological treatments, aging, or gas exchange abnormalities (Gea et al., 2013; Kapchinsky et al., 2018). However, it remains extremely difficult to identify which factor(s) is/are responsible for the alteration of fiber typing. Interestingly, the level of partial pressure of oxygen in the arterial blood (PaO_2_) is correlated with the percentage of type-I fibers in the VL muscle from patients with COPD (Hildebrand et al., 1991). In addition, this latter study showed that the proportion of type-I fibers increased in this muscle 10 days after haemodilution, a method used in COPD patients to reduce hemoglobin concentration in the blood and increase PaO_2_. It was suggested that hypoxemia observed in COPD may result in local muscle hypoxia, which could ultimately affect fiber typing (Hildebrand et al., 1991). However, the degree of hypoxemia is tightly related to the severity of the disease and functional impairment (Whittom et al., 1998). It remains complicated to dissociate the possible effects of hypoxia from that of confounding factors such as chronic inactivity and muscle deconditioning.

**Table 2:** Adaptation of skeletal muscle fiber type under conditions of pathological hypoxia.

**Disease**	**Species**	**Gender**	**Characteristics/model**	**Age**	**Muscles**	**Main effect**	**References**
COPD	Human	M	Stable COPD (predicted FEV1: 62%)	66 y	QUA	↓% FI and ↑% FIIX vs. CT. After haemodilution, ↑% FI and ↓ % FIIX	Hildebrand et al., 1991
		M	Stable COPD (predicted FEV1: <70%)	65 y	VL	↑ % MHC2X vs. CT	Satta et al., 1997
		M, F	Stable COPD (predicted FEV1: 33%)	59 y	DIA	↑ % MHC1 and FI, ↓ % MHC2A, ↓ % MHC2X and FIIX vs. CT	Levine et al., 1997
		M, F	Stable COPD (predicted FEV1: 37%)	65 y	VL	↓ % FI and ↑ % FIIX vs. CT	Whittom et al., 1998
		M	Stable COPD (predicted FEV1: 31%)	65 y	VL	↓ % MHC1 and ↑ % MHC2A vs. CT	Maltais et al., 1999
		M	Stable COPD (predicted FEV1: 55%)	63 y	DEL	X vs. CT	Gea et al., 2001
		M, F	Stable COPD (predicted FEV1: 65%)	65 y	DIA	↑ % FI and ↓ % FIIA vs. CT	Doucet et al., 2004
					VL	X vs. CT	
		M, F	Chronic Obstructive Lung Disease stage II-IV (predicted FEV1: ~40%)	58–67 y	QUA	↑ % FIIX vs. CT	Remels et al., 2007
		M, F	Stable COPD (predicted FEV1: 41%)	68 y	VL	↓ % FI, ↑ % FIIA and ↑ % FIIX vs. CT	Natanek et al., 2013
		M	Chronic Obstructive Lung Disease stage III-IV (predicted FEV1: 26–36%)	66 y	VL	↓ % FI and ↑ % FII vs. CT	Thériault et al., 2014
		M, F	Stable COPD (predicted FEV1: 57 % in nonsarcopenic COPD, 42% in sarcopenic COPD)	Sarcopenic: 68 y; nonsarcopenic: 65 y	VL	↓ % FI, ↑ % FII vs. CT. ↓ % FI in sarcopenic COPD vs. nonsarcopenic COPD	van de Bool et al., 2016
		M, F	Stable COPD (predicted FEV1: 39 % in F, 40 % in M)	F: 63 y; M: 66 y	VL	↓ % FI and ↑ % FII vs. CT	Ausin et al., 2017
		M	Stable COPD (predicted FEV1: 42 % in nonsarcopenic COPD, 29 % in sarcopenic COPD)	Sarcopenic and nonsarcopenic: 65 y	VL	↓ % FI vs. CT. ↑ % FIIA/IIX in sarcopenic COPD vs. CT	Kapchinsky et al., 2018
CHF	Human	M	Coronary artery disease or cardiomaopathy	57 y	GAS	↑ % FIIX vs. CT	Mancini et al., 1989
		M	Left ventricular systolic dysfunction	58 y	VL	↓ % FI and ↑ % FIIX vs. CT	Sullivan et al., 1990
		M	Coronary artery disease, idiopathic dilated cardiomyopathy	56 y	VL	↓ % FI and ↑ % FII vs. CT	Drexler et al., 1992
		M, F	Ischemic heart disease or dilated cardiomyopathy	58 y	VL	↑ % FIIx vs. CT	Schaufelberger et al., 1995
		M, F	Left ventricular systolic dysfunction	48 y	VL	↓ % FI, ↑ % FIIX vs. CT	Lindsay et al., 1996
					DIA	X vs. CT	
					PEC	↓ % FI vs. CT	
					STE	X vs. CT	
		M	Left ventricular systolic dysfunction	61 y	VL	↓ % MHC1 and ↑ % MHC2X vs. CT	Sullivan et al., 1997
		M, F	Dilated cardiomyopathy or coronary artery disease	50 y	DIA	↑ % MHC1 and ↓ % MHC2X vs. CT	Tikunov et al., 1997
		M, F	CHF with preserved ejection fraction	70 y	VL	↓ % FI and ↑ % FII vs. CT	Kitzman et al., 2014
	Mouse		Dilated cardiomyopathy model: deletion mutation K210 in cardiac troponin T gene	2 m	QUA	↓ mRNA levels of *Myh7* and *Myh2*, ↑ mRNA levels of *Myh4* vs. CT	Okada et al., 2015
					SOL	↓ mRNA levels of *Myh7*, ↑ mRNA levels of *Myh4* vs. CT	
	Rat	F	Ligation of the left main coronary artery	Adult	SOL	X vs. CT	Delp et al., 1997
					PLA	↓ % FIIX and ↑ % FIIB vs. CT	
		F	Dahl salt-sensitive rats with high-salt diet	35 w	SOL	X vs. CT	Bowen et al., 2015
					DIA	↑ % FI, ↓ % FIIA vs. CT	
		M	Obese diabetic ZSF1 rats (preserved ejection fraction)	20 w	DIA	↑ % FI, ↓ % FII vs. CT	Bowen et al., 2017
	Minipig	M	Pacing-induced supraventricular tachycardia	6 m	DIA	↑ % FI, ↓ % FIIA vs. CT	Howell et al., 1995
					LD	X vs. CT	
OSAS	Human		Laryngeal carcinoma *in situ* with total laryngectomy	56 y	MPCM	↓ % FI, ↑ % FIIA vs. CT	Ferini-Strambi et al., 1998
					VL	X vs. CT	
		M, F	Recently diagnosed	39 y	TA	Slight ↑ % FIIX and FIIA/IIX vs. CT	Wåhlin Larsson et al., 2008
		M	severe OSAS	40 y	PAL	↓ levels of MHC1	Chen et al., 2016
	Rat		15 s 6–8% O_2_, 10–14% CO_2_ /15 s N, 8 h/ d, 5d/ w for 5 w		GH	↓ % FI and ↑ % FIIB vs. CT	McGuire et al., 2002
					STER	↑ % FI, ↑ % FIIA, ↓ % FIIB vs. CT	
		M	15 s 0 % O_2_/15 s N, 8 h/ d, 5d/ w for 5 w		SOL	X vs. CT	McGuire et al., 2003
					EDL	Slight ↑ % FIIA vs. CT	
		M	240 s 10.3 % O_2_ /240 s N, 7.5 h/ d, for 4 d	Adult	GH	Single fibers: transition from MHC2A to MHC2B	Pae et al., 2005
					DIA	X vs. CT	
					STE	Single fibers: transition from MHC2A/2B to MHC2B	
		M	90 s 5% O_2_ /90 s N, 8 h/d, for 7 d	Adult	STE	X vs. CT	Shortt et al., 2013
					DIA	↓ % FI, ↑ % FIIB vs. CT	
					SOL	X vs. CT	
					EDL	X vs. CT	
		M	4 min 10.3% O_2_/4 min N, 7.5 h/d, for 2 or 4 d	7 w	SOL	X vs. CT	Nguyen et al., 2016
					TA	X vs. CT	

*COPD, chronic obstructive pulmonary disease; CHF, chronic heart failure; OSAS, obstructive sleep apnea syndrome; M, male; F, female; FEV1, forced expiratory volume in the first second; ZSF1, Zucker fatty/spontaneously hypertensive heart failure F1 hybrid; y, year; m, month; w, week; d, day; s, second; N, normoxia; QUA, quadriceps muscle; VL, vastus lateralis muscle; DIA, diaphragm muscle; DEL, deltoid muscle; GAS, gastrocnemius muscle; PEC, pectoralis muscle; STE, sternohyoid muscle; LD, latissimus dorsi muscle; PAL, palatopharyngeus muscle; SOL, soleus muscle; TA, tibialis anterior muscle; EDL, extensor digitorum longus muscle; GH geniohyoid muscle; MPCM, medium pharyngeal constrictor muscle; X, not significantly different; ↑, increase; ↓, decrease; CT, control group; FI, type-I fiber; FIIA, type-IIA fiber; FIIX, type-IIX fiber; FIIB, type-IIB fiber; FIIA/IIX, type-IIA/IIX fiber; MHC1, myosin heavy chain 1; MHC2A, myosin heavy chain 2A; MHC2X, myosin heavy chain 2X; MHC2B, myosin heavy chain 2B; MHC2A/2B, myosin heavy chain 2A/2B; Myh7, myosin heavy chain 7 (encoding MHCI); Myh2, myosin heavy chain 2 (encoding MHCIIA); Myh4, myosin heavy chain 4 (encoding MHCIIB)*.

An opposite adaptation in fiber-type composition is observed in the respiratory muscle diaphragm, compared with the VL muscle (Levine et al., 1997; Doucet et al., 2004). This adaptation is characterized by a larger percentage of type-I fibers and a lower percentage of type-II fibers in patients with COPD than in controls. This fast-to-slow fiber-type transformation is most likely the result of the increased contractile activity of the diaphragm muscle in patients with severe COPD, which is the consequence of the airflow limitation, pulmonary hyperinflation and increased compliance. It is noteworthy that fiber-type adaptation occurs in diaphragm muscle, but not in VL muscle of patients with mild-to-moderate COPD (Doucet et al., 2004). This suggests that the respiratory muscles could show a greater plasticity than limb muscles (Caron et al., 2009), but also that muscle contractile activity (as well as inactivity) is probably a stronger stimulus than other local and systemic factors (such as hypoxia) to modulate fiber-type composition.

### Adaptation of skeletal muscle fiber type in CHF

CHF, a cardiovascular disease highly prevalent in the elderly population, is commonly associated with sarcopenia (Fülster et al., 2013). Similarly to COPD, CHF alters fiber-type composition in both lower/hind limb muscles and diaphragm muscle, and these changes were observed in human and animal studies (Table 2). A reduced proportion of type-I fibers combined with an increased proportion of type-II fibers are commonly found in the VL muscle in patients with CHF compared with controls (Sullivan et al., 1990; Drexler et al., 1992; Lindsay et al., 1996; Kitzman et al., 2014). In addition, muscle biopsies from CHF patients showed a larger percentage of type-IIX fibers in the gastrocnemius muscle and a lower percentage of type-I fibers in the pectoralis muscle compared with control patients, indicating a slow-to-fast fiber-type transition in skeletal muscles with various ranges of function. In contrast, a fast-to-slow shift in fiber-type composition has been reported in the diaphragm muscle from patients with CHF (Tikunov et al., 1997) and in animal models of CHF (Howell et al., 1995; Bowen et al., 2015, 2017). This adaptation is most likely explained by the large contractile activity required by the diaphragm muscle to sustain the increased breathing pattern associated with this disease.

The changes in fiber-type composition presented in the previous paragraph have been reported during both heart failure with reduced ejection fraction (HFrEF) and heart failure with preserved ejection fraction (HFpEF) (for a recent review on skeletal muscle alteration in HFrEF and HFpEF, see Adams et al., 2017). These two forms of CHF are associated with a low tolerance to physical exercise, muscle deconditioning and a reduced quality of life. Interestingly, a positive correlation exists between maximal oxygen uptake and the percentage of type-I fibers in the VL muscle of patients with CHF (Schaufelberger et al., 1995). Several factors have been suggested to explain the changes in fiber-type composition of lower limb muscles observed in CHF patients: inactivity, abnormal hormonal environment, inflammation and malnutrition (Mancini et al., 1989; Schaufelberger et al., 1995). In addition to these factors, the role played by cellular hypoxia in the adaptation of muscle fiber type remains unresolved.

### Adaptation of skeletal muscle fiber type in OSAS

OSAS is a common disorder associated with repeated upper airway collapse during sleep, which leads to disturbed sleep (Jordan et al., 2014). This syndrome is mainly observed in middle-aged individuals, with a large male predominance. Several factors have been associated with OSAS, including anatomical abnormalities of the upper airway, obesity, and smoking (Jordan et al., 2014). The obstruction of the upper airway observed in OSAS also results in hypopnea or apnea, thereby leading to oxygen desaturation and hypoxia. A shift toward a faster profile has been observed in upper airway muscles from patients with OSAS compared with control subjects (Ferini-Strambi et al., 1998; Chen et al., 2016) (Table 2). Ferini-Strambi et al. (1998) reported in the medium pharyngeal constrictor muscle (MPCM) a reduction in the proportion of type-I fibers (30.7 vs. 51.9%) and an increase in the proportion of type-II fibers (61.8 vs. 36.1%) in OSAS patients compared with control subjects (Ferini-Strambi et al., 1998). A similar adaptation was observed in the palatopharyngeus muscle (PAL) from patients with OSAS (Chen et al., 2016). In contrast to the upper airway muscles, changes in fiber-type composition are absent (Ferini-Strambi et al., 1998) or very limited (Wåhlin Larsson et al., 2008) in lower limb muscles from patients with OSAS.

The origin of the higher proportion of fast and more fatigable fibers observed in upper airway muscles from patients with OSAS has been questioned (McGuire et al., 2002). To elucidate whether this adaptation is a consequence (and not a cause) of OSAS, animal studies have been developed. Chronic intermittent hypoxia (CIH), which accompanies OAS as a result of repetitive apnoeic periods, is a well-established model in rodents to mimic this pathological condition. Analysis of the fiber-type composition revealed a slow-to-fast fiber-type transition in the geniohyoid (GH) muscle of rats exposed to CIH, with changes already observed after a few hours of exposure (Pae et al., 2005) and maintained after 5 weeks (intermittent exposure 8 h/day) (McGuire et al., 2002) (Table 2). The results observed in another upper airway dilator, the sternohyoid muscle (STE), are more controversial. A transition from MHC2A/2B to MHC2B has been reported in single fibers from the STE muscle of rats exposed to a 20 h-period of intermittent hypoxia (Pae et al., 2005). A lower proportion of type-IIB fibers, as well as a larger proportion of type-I and type-IIA fibers were found in this muscle after 5 weeks (intermittent exposure 8 h/day) (McGuire et al., 2002), while no changes in the fiber typing were observed after 7 days of intermittent hypoxia (8 h/day) (Shortt et al., 2013). Changes in the fiber-type composition of the diaphragm muscle in response to CIH are also not univocal, with no changes (Pae et al., 2005) or a slow-to-fast fiber transition observed (Shortt et al., 2013). The inconsistency between the results could be explained, among other factors, by the specificity of the protocols used (pattern and duration of the cycles, total duration of exposure, level of hypoxia, presence of hypercapnic hypoxia, etc.). Finally, the fiber-type composition in hind limb muscles remained unchanged (McGuire et al., 2003; Shortt et al., 2013; Nguyen et al., 2016) or only slightly affected (McGuire et al., 2003) in response to CIH in rats.

The mechanisms responsible for the slow-to-fast fiber-type transition observed in the upper airway muscles of patients with OSAS and in rats exposed to CIH remain uncertain. An increased sympathetic outflow has been reported in patients with OSAS, and this could impair muscle sympathetic nerve tone in humans (Xie et al., 2001). An increased sympathetic outflow may result in a slow-to-fast muscle fiber-type shift, as observed in soleus muscles from rats treated with the β2-adrenergic agonist clenbuterol (Bricout et al., 2004). As previously hypothesized (Pae et al., 2005), CIH occurs during sleep-disordered breathing, and may rapidly influence the central nervous system by altering sympathetic and somatic motor outflows, thereby contributing to muscle fiber-type adaptation. More recently, it was proposed that the downregulation of estrogen-related receptor α (ERRα) could be a factor responsible for the reduced expression of MHC-I in palatopharyngeal muscle from patients with OSAS (Chen et al., 2016).

## Hypoxia-inducible factor-1 signaling pathway as a modulator of skeletal muscle fiber-type composition

One of the major adaptive responses to low oxygen availability at the cellular level is conferred by activation of the hypoxia-sensitive transcription factor hypoxia-inducible factor-1 (HIF-1) [for review, the reader could refer to (Semenza, 2010)]. HIF-1 is composed of two subunits, a constitutively expressed HIF-1β subunit and an O_2_-sensitive HIF-1α subunit. HIF-1β and HIF-1α proteins are expressed in most tissues, including skeletal muscle, but HIF-1α is unstable in the presence of oxygen and becomes hydroxylated and degraded. Under hypoxic conditions, HIF-1α accumulates and translocates into the nucleus where it forms the heterodimeric protein HIF-1 with HIF-1β, thereby leading to the activation of target genes involved in several biological processes, including angiogenesis, oxygen transport and energy metabolism.

### HIF-1α expression and skeletal muscle fiber-type specificity

Several murine studies indicate that HIF-1α protein is more abundant in glycolytic muscles than in oxidative muscles under normoxic conditions (Pisani and Dechesne, 2005; Lunde et al., 2011; Nguyen et al., 2016). In contrast, a higher expression of HIF-1α protein has been reported in predominantly oxidative muscles than in predominantly glycolytic muscles in human subjects (Mounier et al., 2010). HIF-1α mRNA levels were lower in oxidative than in glycolytic muscles in both mice (Pisani and Dechesne, 2005) and humans (Mounier et al., 2010), suggesting that the divergence in HIF-1α protein content between murine and human studies may result from differences in post-transcriptional and/or post-translational mechanisms.

### Role played by the HIF-1 signaling pathway in the determination of skeletal muscle fiber type

The involvement of HIF-1 signaling pathway in the regulation of skeletal muscle fiber type has been investigated but the results are not univocal. One study evaluated the composition of muscle fiber type in mice with a skeletal muscle-specific deletion of HIF-1α (Mason et al., 2004). It was shown that the proportion of type-IIA fibers was slightly reduced in soleus muscles from HIF-1α knockout mice compared with wild-type mice (i.e., suggesting a slightly slower fiber-type profile), while the fiber-type composition was not different in the gastrocnemius muscles between these two groups. Interestingly, this study revealed that endurance performance was higher in untrained HIF-1α knockout mice than in wild-type mice. However, repeated exercise bouts induced severe muscle damage in these knockout mice, which led to an impaired running performance after 4 consecutive days of strenuous exercise training. A follow-up study from the same group demonstrated that HIF-1α deletion in skeletal muscle blunted the fast-to-slow fiber-type shift and the enhancement of oxidative capacity following 6 weeks of endurance training (Mason et al., 2007). Since these HIF-1α null mice have already undergone adaptation before training, it was proposed that additional endurance training was unable to further improve muscle adaptation. In a more recent study, Lunde et al. (2011) observed a slow-to-fast fiber-type transformation in both the EDL and soleus muscles of rats when HIF-1α was overexpressed by electroporation (Lunde et al., 2011). In addition, this study showed that the proportion of MHC2B mRNA increased whereas that of MHC1 decreased in C2C12 myotubes transfected with HIF-1α, a result which is in agreement with the histochemical analysis performed on skeletal muscle fibers.

Another study has recently investigated the impact of prolyl hydroxylase domain 2 protein (PHD2) invalidation on skeletal muscle fiber-type distribution in mice (Shin et al., 2016). PHD2, a member of the PHD family, is an enzyme that hydroxylates HIF-1α, which is then ubiquitinated and degraded by the proteasome under normoxic conditions. This study showed that PHD2 deletion induced HIF-1α protein stabilization in skeletal muscle, and led to a fast-to-slow fiber-type shift in the soleus and gastrocnemius muscles. It was suggested that this contractile adaptation may be the result of the activation of the calcineurin/nuclear factor of activated T cell 1 (NFATc1) pathway. In addition to HIF-1α, HIF-2α is also able to accumulate in skeletal muscle from these PHD2 null mice (Nunomiya et al., 2017), and the deletion of HIF-2α led to a slow-to fast fiber-type transition in murine soleus muscles (Rasbach et al., 2010). Therefore, the possibility that HIF-2α has contributed to the muscle fiber-type shift toward a slower phenotype in the PHD2 null mice in the later study (Shin et al., 2016) cannot be excluded. Recently, Nunomiya et al. showed that PHD2 null mice displayed an enhanced endurance performance after running training compared with control mice (Nunomiya et al., 2017) but unfortunately the adaptation of muscle fiber-type composition was not investigated in this study.

Altogether, some evidences indicate that HIF-1α promotes a slow-to-fast fiber-type transition in skeletal muscle. We believe that HIF-1α and HIF-2α could have distinct roles in the determination of contractile phenotype, and results from studies using genetically modified animal models suggest that HIF-1α and HIF-2α could be connected with other signaling pathways.

## Implication of cellular hypoxia and HIF-1α in the adaptation of skeletal muscle fiber type to high-altitude exposure and in COPD, CHF and OSAS

**Figure 1:**
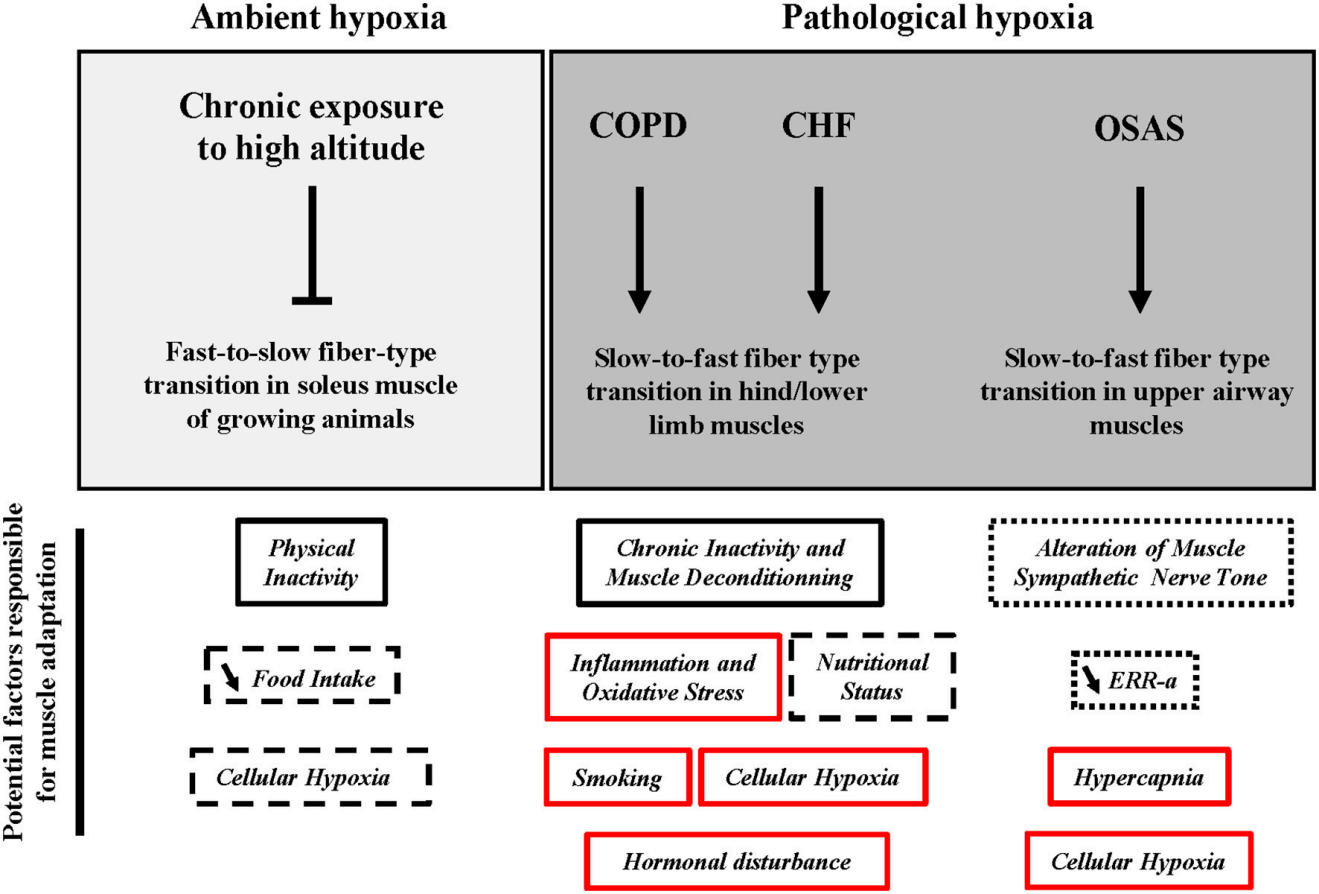
Potential factors responsible for the adaptation of skeletal muscle fiber type in response to chronic exposure to high altitude and under conditions of pathological hypoxia. COPD, chronic obstructive pulmonary disease; CHF, chronic heart failure; OSAS, obstructive sleep apnea syndrome; ERR- α, estrogen related receptor-alpha. Vertical arrow, activation; Vertical blunt arrow, inhibition; ↘, decrease. 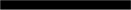
: Factor most likely responsible. 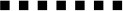
: Factor possibly responsible. 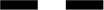
: Factor most unlikely responsible. 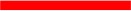
: Role played unclear or unknown.

This review has presented the idea that chronic exposure to high altitude leads to the impairment of the fast-to-slow fiber-type shift in soleus muscles from rats during post-natal development. In addition, a slow-to-fast fiber-type shift is observed in the lower limb muscles during COPD and CHF and in upper airway muscles during OSAS. Several factors have been proposed to explain this shift toward a faster fiber-type profile and a summary is presented in Figure 1. This part will discuss the potential implication of cellular hypoxia and HIF-1α in this muscle adaptation.

The first question that arises is whether cellular hypoxia is observed in skeletal muscle during exposure to high altitude or under conditions of pathological hypoxia. A lower intracellular oxygen partial pressure (PiO_2_) has been detected in human skeletal muscle at rest in acute hypoxic conditions (ambient air with 10% O_2_, corresponding to a simulated altitude of 5,800 m) than in normoxic conditions (ambient air with 20.9% O_2_) (PiO_2_ = 23 mmHg and 34 mmHg, respectively) (Richardson et al., 2006). In mice exposed to an identical level of hypoxia (ambient air with 10% O_2_), PiO_2_ in skeletal muscle was reduced from ~50 to 20 mmHg (Reinke et al., 2011). Interestingly, intramuscular PiO_2_ observed in humans decreased to ~3 mmHg at intensities above 50% of maximal oxygen uptake under normoxic conditions, a result certainly explained by the higher mitochondrial respiration (Richardson et al., 1995). These results indicate that hypoxic stress at the intramuscular level remains moderate during acute exposure to severe ambient hypoxia, and is substantially larger during a moderately intense endurance exercise. To date, it remains unknown whether a reduction of intramuscular PO_2_ still persists after chronic exposure to high altitude. A reduction of PaO_2_ is commonly observed in patients with severe COPD (Hildebrand et al., 1991), CHF (Mirzaaghazadeh et al., 2014), and OSAS (Xie et al., 2016), but to our knowledge, intramuscular PO_2_ has never been investigated in these populations.

As described before, HIF-1α is well-known to be the master regulator of the cellular adaptive response to hypoxic stress (Semenza, 2010). In addition, HIF-1α seems to play a role in the determination of skeletal muscle fiber type through a slow-to-fast transition. Could this hypoxia-sensitive factor be involved in the adaptation of hind/lower limb muscle fiber type in response to high-altitude exposure in growing animals and under conditions of pathological hypoxia? To date, results showing an increased stabilization of HIF-1α protein in skeletal muscle after exposure to high altitude, are not evident in humans (Viganò et al., 2008), and are usually found in animals only after exposure to severe altitude (normobaric or hypobaric hypoxia corresponding to a simulated altitude >5,800 m) (Stroka et al., 2001; De Palma et al., 2007; Chaudhary et al., 2012). In their study, Chaudhary et al., observed that HIF-1α protein levels increased in the gastrocnemius muscle from rats exposed to a simulated altitude of 7,620 m, with a maximum increase observed after 14 days of exposure (Chaudhary et al., 2012). They also found that the production of reactive oxygen species (ROS) and oxidative stress, evaluated from free radical generation and lipid peroxidation, followed a similar profile (i.e., increase) during chronic exposure to severe hypoxia, suggesting that ROS may also be a key signal for stabilizing HIF-1α protein. In addition to the possibility that skeletal muscles are low responders to reduced O_2_ delivery, technical limitations could explain the difficulty to detect hypoxia-induced HIF-1α protein stabilization in skeletal muscle; these limitations include the short half-life of HIF-1α, which can potentially reduce its content during tissue collection, as well as the technical complexity of accurately detecting HIF-1α from immunoblot analysis. Unfortunately, none of the studies presenting an impairment of the fast-to-slow fiber-type shift in growing rats have assessed HIF-1α protein levels. However, since HIF-1α protein does not seem to accumulate at an altitude of 4,000 m, (i.e. the altitude used in several of these studies, see Table 1), it is hypothesized that cellular hypoxia and HIF-1α do not play a major role in this muscle adaptation.

The regulation of HIF-1α is not well-documented in skeletal muscle from patients with COPD, CHF and OSAS. One study observed a tendency to an increase in HIF-1α mRNA in skeletal muscle from patients with COPD, but unfortunately, HIF-1α protein content was not determined (Jatta et al., 2009). In a murine model of OSAS, HIF-1α protein was not detectable in the gastrocnemius muscle in response to 14 days of CIH, while its content in both the liver and the epididymal adipose tissue was higher in the intermittent hypoxic conditions than in normoxia (Thomas et al., 2017). Furthermore, HIF-1α mRNA and protein levels increased in the genioglossus muscle (i.e., a muscle of the tongue) from rats exposed to 35 days of CIH (Jia and Liu, 2010), while HIF-1α protein content in the soleus muscle was not affected after 28 days of CIH, and was even reduced after 35 days of CIH (Sacramento et al., 2016). This latter study also found that HIF-1α accumulated in the liver after CIH. These findings suggest that HIF-1α protein accumulation is tissue-specific and also certainly muscle-specific in response to intermittent hypoxia. In addition, it is important to keep in mind that CIH stimulates the production ROS in skeletal muscle, which also promotes the accumulation of HIF-1α protein (Yuan et al., 2008). Since ROS and oxidative stress also seem to increase in skeletal muscle during COPD (Gea et al., 2013) and CHF (Martinez et al., 2015), we believe that HIF-1α protein could possibly accumulate in these three pathologies. To date, the role of cellular hypoxia and HIF-1α in the adaptation of muscle contractile phenotype in COPD, CHF, and OSAS is unclear and needs to be further investigated.

## Changes in expression of molecular regulators of fiber-type composition under hypoxic conditions

Skeletal muscle fibers have different properties with respect to force, shortening velocity, time of relaxation, fatigability, and metabolic capacities. These properties determine the muscle phenotype and profile and can be modulated by several stimuli, as explained in the introduction. This review provides evidences that the changes in fiber-type composition observed in hind/lower limb skeletal muscles under hypoxic conditions (high altitude in growing animals, and under conditions of pathological hypoxia such as CHF and COPD in both human and animal studies) could be mainly the result of physical inactivity and muscle deconditioning. The pattern of electrical activity during muscle contraction appears to be an essential signal responsible for fiber-type transformation and changes in the composition of contractile proteins such as MHC (for review, see Gundersen, 2011). Several signals have been proposed to serve as messengers detected by molecular sensors, which then trigger intracellular signaling cascades, thereby leading to changes in gene expression programs related to fiber-type profile (Gundersen, 2011). Some important molecular regulators of fiber-type composition showing changes in gene expression in skeletal muscles under the hypoxic conditions presented above will be discussed in this section.

### Calcineurin/NFAT pathway

The electrical stimulation pattern modulates the level of intracellular calcium (${\text{Ca}}_{\text{i}}^{2+}$). Moderate but prolonged increases in ${\text{Ca}}_{\text{i}}^{2+}$ concentration have been shown to activate the phosphatase calcineurin, which leads to the dephosphorylation and nuclear translocation of nuclear factor of activated T-cells (NFAT) proteins. NFAT proteins can then bind to specific promoters in conjunction with other transcriptional regulators, including myocyte enhancer factor 2 (MEF2), resulting in the transcription of genes encoding proteins of the slow fiber program (Chin et al., 1998).

We recently assessed the mRNA level of regulator of calcineurin 1 (Rcan1, called Mcip1 for myocyte-enriched calcineurin-interacting protein 1 in these studies), an indirect marker of calcineurin activity (Chaillou et al., 2013, 2014). The mRNA level of Rcan1 was increased in both plantaris and soleus muscles after 9 and 10 days of ambient hypoxia, respectively. However, these changes were only transient and do not corroborate with the absence of fiber-type transformation observed after several weeks of hypoxia exposure.

In a human study, a higher level of calcineurin protein was observed in the VL muscle from patients with COPD compared with control patients (Natanek et al., 2013). This result is surprising due to the fact that these COPD patients displayed a slow-to-fast fiber-type shift. It is important to notice that the activity of calcineurin was not evaluated in this study, and the mRNA level of RCAN1 did not reveal any differences between these two groups. Similarly, RCAN1 mRNA level in the VL muscle was not different between patients with CHF and controls (Garnier et al., 2005). Furthermore, a reduced content of calcineurin protein was found in the soleus muscles from rats with CHF compared with controls, and this result was consistent with a shift toward a faster fiber-type profile (Vescovo et al., 2005). To date, the exact role played by calcineurin in skeletal muscle under hypoxic conditions needs to be further investigated.

### PGC-1α and PPARδ

Peroxisome proliferator-activated receptor γ (PPARγ) coactivator-1α (PGC-1α) is a co-activator of transcription factors, including peroxisome proliferator-activated receptor δ (PPARδ). In addition to their role in regulating pathways related to oxidative capacity and lipid metabolism (Gundersen, 2011), these two factors have been implicated in the fast-to-slow fiber-type transformation (Lin et al., 2002; Lunde et al., 2007).

Although changes in fiber-type composition were found, chronic exposure to severe normobaric hypoxia did not affect PGC-1α protein level in skeletal muscle from young mice (Slot et al., 2016; O'Brien et al., 2018). In contrast, we observed a slight decrease in PGC-1α protein content in the plantaris muscle from young rats after 9 days of hypobaric hypoxia, while an opposite result was observed at the mRNA level at this time point (Chaillou et al., 2013). Pgc-1α mRNA level in the soleus muscle was also higher in young rats exposed 14 days to severe hypobaric hypoxia compared with control animals (Chaillou et al., 2014), but no changes in fiber type were found in this study. Recently, a reduced content of PGC-1α protein was found in skeletal muscle after 66 days in high altitude in humans, a result consistent with a decreased mitochondrial density (Levett et al., 2012). Even if the composition of fiber-type was not assessed in this study, it is very unlikely that a slow-to-fast shift would have been observed due to the fact that the participants were subjected to extensive physical activity during the expedition.

PGC-1α mRNA levels, as well as PPARδ protein content were reduced in the quadriceps of patients with COPD, a result that corroborates with the increased proportion of type-IIX fibers in these patients (Remels et al., 2007). In a rat model of CHF, PGC-1α protein content was reduced in soleus muscles compared with controls, and again, this result was consistent with the fast-to-slow fiber-type transition observed (Vescovo et al., 2005). However, PGC-1α mRNA levels were not reduced in skeletal muscle from patients with CHF (Garnier et al., 2005).

Altogether, some studies suggest that PGC-1α expression is affected during exposure to ambient hypoxia, but this cofactor does not seem to play a major role in the modulation of fiber type in this hypoxic condition. Some evidences indicate that the impaired content of PGC-1α and PPARδ, in addition to potentially explain the disturbed muscle oxidative capacity, may contribute to the slow-to-fast fiber-type transition in COPD patients. Due to the inconsistent results between animal and human studies on CHF, the exact role of these two regulatory factors remains elusive in this disease.

### miR-499/208b

Micro-RNAs (miRs) are small non-coding RNAs that regulate gene expression through a posttranscriptional mechanism. Several miRs are expressed specifically in striated muscle, such as miR-1, miR133a, miR-208b, miR-486 and miR-499, while miR-208a and miR-206 are specifically expressed in cardiac muscle and skeletal muscle, respectively (McCarthy, 2011). A previous study has demonstrated that miR-499 and miR-208b overexpression induce the activation of the slow myofiber gene program, partly through the suppression of SOX6, a member of the SOX (Sry-related high motility group) family (van Rooij et al., 2009). In addition, it has been recently demonstrated that the transcription factors PPARβ/δ promote the expression of the estrogen-related receptor γ (ERRγ), which activates the transcription of miR-499 and miR-208b, thereby driving the slow fiber program (Gan et al., 2013).

A recent study in a rat model of COPD suggests that the reduced levels of miR-208b and miR-499, associated with an increased content of SOX6 protein, may contribute to the faster fiber-type profile observed in both the soleus and gastrocnemius muscles (Huang et al., 2016). A trend to a lower level of miR-499 was found in quadriceps muscle from COPD patients compared with control patients (Lewis et al., 2012). In contrast, circulating miR-499 level was higher in a cohort of COPD patients than in matched controls (Donaldson et al., 2013). Interestingly, this latter study showed that circulating miR-499 was lower in COPD patients with a pathological fiber shift (i.e., slow-to-fast shift) than in COPD patients who did not display any changes in fiber-type composition. It is noteworthy that miR-499 level was not assessed in skeletal muscle in this study, which makes difficult to draw any clear conclusions. Recently, the expression profiling of 373 mature miRs was determined in soleus muscles in a rat model of CHF (Moraes et al., 2017). A slow-to-fast fiber-type transformation was observed in this model, but this result was not paralleled with changes in miR-499 and miR-208b levels.

To summarize, miR-208b and miR-499 may be two candidates that could explain the changes in fiber-type composition of hind/lower limb muscles under pathological hypoxia, at least during COPD, but the regulation of these muscle-specific miRs needs to be further investigated in hypoxic conditions. Interestingly, low frequency electrical stimulation increases the number of type-I fibers in a rat model of COPD, a result associated with an increased expression of mir-499 and miR-208b and a decreased content of SOX6 (Huang et al., 2016). Thus, it is possible that low frequency electrical stimulation could be beneficial to improve muscle function and facilitate the recovery of a normal fiber-type profile in COPD patients with exercise intolerance. These miRs show promise for the identification of therapeutic targets aimed at maintaining muscle function in chronic diseases, such as COPD and CHF.

## Conclusion

In summary, the majority of the studies indicates that chronic exposure to high altitude does not markedly affect the fiber-type composition of hind/lower limb skeletal muscles in both adult animals and humans, at least when it is not combined with intense physical activity. An alteration of the fast-to-slow fiber-type transition is observed in the soleus muscle during post-natal development in growing rats exposed to severe altitude, and this adaptation is most likely the result of the reduced locomotor activity consecutive to hypoxia exposure. A slow-to-fast shift in fiber type is a common feature in lower limb muscles from patients with COPD and CHF. Several factors have been proposed to explain the dysfunction of lower limb muscles in these two pathologies, with chronic inactivity and muscle deconditioning being most likely the main factors responsible for these changes. In contrast, a transition toward a slower fiber-type profile is often described in the diaphragm muscle in COPD and CHF, and this adaptation appears to result from the increased contractile activity of this respiratory muscle. Most of the studies investigating the adaptation of fiber type during OSAS has been performed in the animal model of CIH. These studies reveal a transition toward a faster profile in the upper airway muscles, while the fiber-type composition is likely not affected in hind limb muscles. Some hypotheses have been identified to explain the modulation of fiber type in the upper airway muscles during OSAS, including an alteration of muscle sympathetic nerve tone and a downregulation of ERR-α expression in skeletal muscle. Some evidences from genetically modified models indicate that the hypoxia-sensitive factor HIF-1α promotes a slow-to-fast fiber-type transition in skeletal muscle; however, the ability of HIF-1α to accumulate in skeletal muscle in response to a hypoxic stimulus is uncertain. We propose that cellular hypoxia and HIF-1α are not involved in alteration of the fast-to-slow shift in soleus fiber type from growing rats exposed to severe altitude, while their role in the adaptation of muscle contractile phenotype in COPD, CHF, and OSAS remains to be elucidated. Finally, we have identified PGC-1α and PPARδ, as well as miR-499 and miR-208b as potential targets to improve muscle function and facilitate the recovery of a normal fiber-type profile in patients with COPD.

## Author contributions

The author confirms being the sole contributor of this work and has approved it for publication.

### Conflict of interest statement

The author declares that the research was conducted in the absence of any commercial or financial relationships that could be construed as a potential conflict of interest.
